# Artificial intelligence and endoanal ultrasound: pioneering automated differentiation of benign anal and sphincter lesions

**DOI:** 10.1007/s10151-025-03160-0

**Published:** 2025-06-10

**Authors:** M. Mascarenhas, M. J. Almeida, M. Martins, F. Mendes, J. Mota, P. Cardoso, B. Mendes, J. Ferreira, G. Macedo, C. Poças

**Affiliations:** 1 Department of Gastroenterology, Precision Medicine Unit, São João University Hospital, 4200-427 Porto, Portugal; 2 WGO Gastroenterology and Hepatology Training Center, 4200-047 Porto, Portugal; 3 Faculty of Medicine of the University of Porto, 4200-047 Porto, Portugal; 4 Department of Mechanical Engineering, Faculty of Engineering of the University of Porto, 4200-065 Porto, Portugal; 5 Manoph Gastroenterology Clinic, 4000-007 Porto, Portugal; 6 School of Medicine and Biomedical Sciences (ICBAS), 4050-313 Porto, Portugal

**Keywords:** Anal fissure, Anal laceration, Artificial intelligence, Endoanal ultrasound

## Abstract

**Background:**

Anal injuries, such as lacerations and fissures, are challenging to diagnose because of their anatomical complexity. Endoanal ultrasound (EAUS) has proven to be a reliable tool for detailed visualization of anal structures but relies on expert interpretation. Artificial intelligence (AI) may offer a solution for more accurate and consistent diagnoses. This study aims to develop and test a convolutional neural network (CNN)-based algorithm for automatic classification of fissures and anal lacerations (internal and external) on EUAS.

**Methods:**

A single-center retrospective study analyzed 238 EUAS radial probe exams (April 2022–January 2024), categorizing 4528 frames into fissures (516), external lacerations (2174), and internal lacerations (1838), following validation by three experts. Data was split 80% for training and 20% for testing. Performance metrics included sensitivity, specificity, and accuracy.

**Results:**

For external lacerations, the CNN achieved 82.5% sensitivity, 93.5% specificity, and 88.2% accuracy. For internal lacerations, achieved 91.7% sensitivity, 85.9% specificity, and 88.2% accuracy. For anal fissures, achieved 100% sensitivity, specificity, and accuracy.

**Conclusion:**

This first EUAS AI-assisted model for differentiating benign anal injuries demonstrates excellent diagnostic performance. It highlights AI’s potential to improve accuracy, reduce reliance on expertise, and support broader clinical adoption. While currently limited by small dataset and single-center scope, this work represents a significant step towards integrating AI in proctology.

## Introduction

The anal canal is a determinant structure for maintaining fecal continence, with external anal sphincter (EAS) and internal anal sphincter (IAS) playing a critical role in maintaining a resting anal tone [1].

Benign anorectal disorders, including anal fissures and sphincteric lacerations, are highly prevalent across both sexes and all age groups. These conditions, while not life-threatening, can profoundly impact a patient’s quality of life. Therefore, an accurate diagnosis is crucial to relieve symptoms, prevent complications, and improve patient outcomes [1, 2].

Although benign anorectal disorders are usually multifactorial and can occur with intact anal sphincters, structural anal sphincteric disease remains a major contributing factor [1]. The cause of such injuries is highly variable, encompassing obstetric injury during vaginal delivery [3, 4], anorectal surgeries, hemorrhoidectomy, anal penetration, rectal prolapse, altered bowel habits, radiation toxicity, among others [1, 5, 6].

Endoanal ultrasonography (EAUS) is the current gold standard for evaluating sphincteric integrity and a key factor attributing a structural causality to proctologic conditions, including asymptomatic women with obstetric injuries. It is a simple, well tolerated, and inexpensive technique, performing better than magnetic resonance for IAS defects (appearing as an interruption in the hypoechoic ring) and equivalent for EAS defects (represented as a break, usually hypoechoic in the normal texture of the echogenic ring) [1, 7, 8].

Nevertheless, EAUS has some noteworthy limitations: a relatively steep learning curve, requiring extensive training and practice to achieve proficiency; limited accessibility, often restricted to specialized centers. As a result, only a small number of professionals are capable of employing it systematically and effectively, which restricts its widespread application and integration into routine practice (e.g., integration into routine proctologic evaluations) [8]. Furthermore, the technique is also affected by a considerable intra- and intervariability, which can hinder the training process and the reproducibility of its results. These challenges exacerbate the consequences of limited access to the technique, further impeding its adoption as a standard tool in clinical practice.

Artificial intelligence (AI), much like its use in other imaging-based diagnostic methodologies within gastroenterology, holds significant potential to enhance diagnostic accuracy and support clinicians in EAUS. It not only addresses the scarcity of specialized professionals but also mitigates the challenges posed by the long learning curve required to achieve proficiency in this technique.

This article aims to present a proof-of-concept model for the automatic identification and characterization of morphologic patterns associated with common benign anorectal conditions in EAUS, particularly anal lacerations and fissures.

## Methods

A total of 238 three-dimensional (3D) endoanal ultrasound (EAUS) procedures conducted at the ManopH Gastroenterology Clinic (Porto, Portugal) between April 2022 and January 2024 were analyzed. All procedures were performed by a highly experienced EAUS gastroenterologist, using a General Electric (GE) Healthcare Flex Focus 500 ultrasound system.

The collected raw data from each endoanal ultrasound procedure was processed for subsequent labeling, ensuring patient confidentiality and complete anonymization before further analysis. Frames were independently classified by two gastroenterologists. In cases where no consensus was reached, a third expert reviewer was consulted. Only frames with agreement among clinicians were included. The dataset comprised a total of 4528 lesion-containing frames. These were categorized into fissures (516), external lacerations (2174), and internal lacerations (1838).

**Fig. 1:**
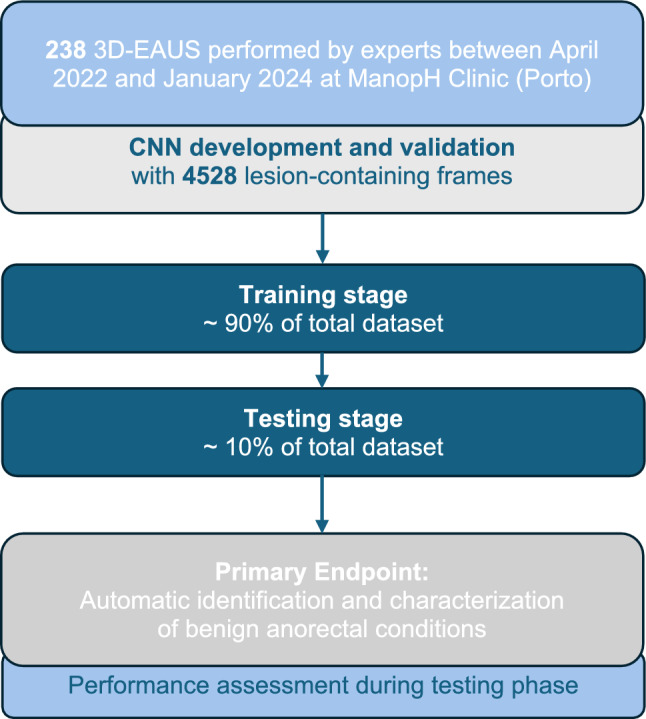
Study flowchart for the training and testing stages

A convolutional neural network (CNN) was developed for automatic classification of fissures and anal lacerations (external and internal). We used 90% (*n* = 4075) of the data to train and validate the model (allocating 90% for training and 10% for hyperparameter fine-tuning validation), while the remaining 10% (*n* = 453) was set aside for independent testing. Procedural split between training and testing sets was not implemented to maximize the use of data, opting instead for lesion-level stratification. A graphical flowchart of the study design is shown in Fig. 1.

The CNN was developed using the latest YOLOv11 (You Only Look Once) classification model, which offers improved accuracy over previous versions while using fewer parameters (1.6 million). The model was pre-trained on the ImageNet-1k dataset, a large multi-categorical image dataset created at Stanford University. In classification mode, YOLO outputs a single class label and a confidence score. The training was conducted in a system with a dual NVIDIA Quadro RTXTM 80000 GPUs (NVIDIA Corp, Santa Clara, CA, USA) and an Intel 2.1 GHz Xeon Gold 6130 processor (Intel, Santa Clara, CA, USA) for 100 epochs to ensure effective convergence of the model and balance performance and computational efficiency. A batch size of 32 was used, images were resized to 256 × 256, and the initial learning rate was set to 0.01. The AdamW optimizer, a stochastic optimization method, was employed. This method modifies the standard weight decay implementation in Adam by decoupling the weight decay from the gradient update. This retrospective study had no influence on patient management because of its non-interventional design.

## Results

In total, 238 EAUS exams were conducted between April 2022 and January 2024 at ManopH Clinic (Porto, Portugal). We included 4528 lesion-containing frames for the development of this CNN, of which 516 corresponded to fissures, 2174 corresponded to external lacerations, and 1838 corresponded to internal lacerations.

The model was trained and developed using 90% of the total dataset (*n* = 4075). The remaining 10% was used for independent testing. Table 1 shows the confusion matrix between the CNN’s predictions in test set versus the expert classification, considered the gold standard.

**Table 1:** Confusion matrix of the automatic detection versus final diagnosis; CNN: convolutional neural network

CNN classification	Final diagnosis		
	Anal fissure	External laceration	Internal laceration
Anal fissure	52	0	0
External laceration	0	179	15
Internal laceration	0	38	165

**Fig. 2:**
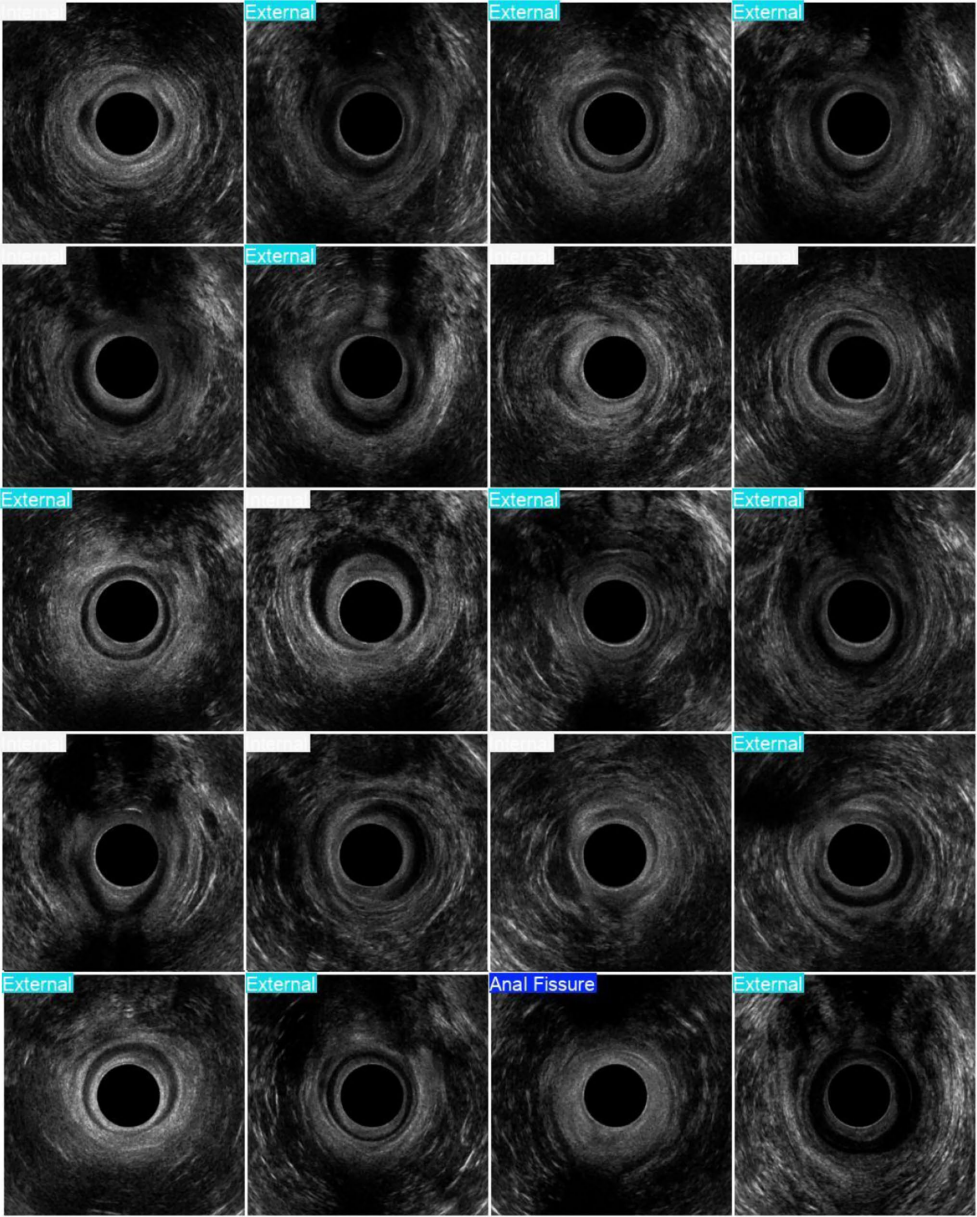
Examples of the output provided by the model. External–external anal laceration; internal–internal anal laceration

For external lacerations, sensitivity, specificity, and accuracy were 82.5%, 93.5%, and 88.2%, respectively. For internal lacerations, sensitivity was 91.7%, specificity 85.9%, and accuracy 88.2%. Notably, for anal fissures, the model achieved 100% sensitivity, specificity, and accuracy (Fig. 2).

## Discussion

The present study represents a pioneering effort in evaluating the application of AI deep learning methods for the automatic detection of benign anal lesions in endoanal ultrasound images, serving as a proof-of-concept and foundation for expanding the role of AI in the field of proctology.

In the literature, there are previous attempts to develop deep learning systems for the detection of malignant pathology in the rectum using EAUS images, with sustainable accuracy in detecting and differentiating it from normal rectal images [9].

The integration of AI into EAUS holds significant promise, not only because it is recognized as the current gold standard for assessing the sphincteric complex but also as a result of challenges hindering its optimal implementation, namely limited widespread availability, a steep learning curve, and a relatively small number of highly proficient practitioners.

From a technical perspective, performing EAUS with an automated 360° transducer is relatively straightforward; however, image interpretation is the limiting step, as it is demanding and requires expertise. Therefore, AI has the potential to play a transformative role in medical education and training in this diagnostic modality. By providing real-time guidance and automated visual feedback, AI can support the learning process of less experienced professionals in EAUS, enabling them to acquire and refine their skills more efficiently. This approach complements the traditional reliance on a second expert opinion while ensuring a “human-in-the loop” framework, preserving the critical role of human oversight in clinical decision-making.

Although anal fissures are typically diagnosed through clinical evaluation and physical examination, and the utility of EAUS for this purpose is limited, their inclusion in this study was considered an added value and a way to enrich the model with multiple and common benign pathologies.

While EAUS is not the standard modality for diagnosing anal fissures, these findings may contribute to the future development of broader AI-assisted models capable of recognizing a variety of anorectal pathologies, even when not specifically sought during the examination. In this context, the satisfactory though preliminary results achieved in the classification of benign anal lesions, namely anal lacerations and fissures, offer a promising foundation for future efforts focused on the detection and differentiation of pleomorphic lesions affecting the anal sphincteric complex, with the ultimate goal of enhancing diagnostic accuracy, guiding therapeutic decisions, and improving outcomes in proctologic care.

There are some limitations that should be mentioned. First, the study is retrospective and unicentric, which introduces a selection bias and may affect the external validity of the results. Additionally, the model was developed using still frames, which could limit its effectiveness when applied in real-world scenarios. It is also important to acknowledge that there are some other anal pathologies that add complexity to EAUS interpretation and have not yet been addressed. Nonetheless, this paper is worth sharing since the novelty of this AI model lies in its role as a proof of concept, demonstrating that AI can also be applied and potentially have a transformative impact in EAUS.

The use of a YOLO CNN may also enable AI-provided real-time feedback to proctologists during the assessment of the anal canal in the near future, as more robust versions of this AI model are developed. It will help highlight areas with the highest probability of containing a lesion. This explainable AI approach is particularly valuable given the black box nature of this algorithm, as it plays a key role in advancing the development of trustworthy AI for clinical practice [10].

This dual role of improving diagnostic precision while fostering professional development underscores the transformative potential of AI in revolutionizing the field of endoanal ultrasound and proctology. AI may be a potential teaching tool, providing practitioners, especially those just starting out in their careers, with real-time guidance, automated feedback, and individualized learning opportunities. Ultimately, the application of AI in endoanal ultrasound could improve clinical outcomes, increase access to expertise, and drive innovation in the proctology field.

## Data Availability

Data is provided within the manuscript or supplementary information files.
